# To Live Long

**Published:** 1855-01

**Authors:** 


### HALL'S JOURNAL OF HEALTH.

VOL. II.] JANUARY, 1855. [NO. I.

### TO LIVE LONG.

To-day we enter upon a new year, but before its close, many an eye which now sparkles in gladness, will have fallen into the sleep which knows no waking; many a cheek which bears upon itself the bloom of health, will have paled away into the iciness of death; many a manly form, defiant of sickness now, will have lain itself in the last resting-place of all; and many a strong heart which is this day flushed with the successes of life, and by these successes has grown boundless in its ambitions, will have wasted and worn itself away in its fruitless struggles with unexpected disease. Half of all who are to die during eighteen hundred and fifty-five, will not have reached the age of twenty-one years while they ought to reach the full limit of three score and ten. Our Maker has constructed the human machine to work easily, healthfully and well for seventy years, and that is the period which he has appointed to us, and which he has guaranteed to us, on the condition of a life of temperance, wisdom, and piety. Why is it that of the nine millions of human beings who, as the venerable and distinguished President Nott told us last night, in his eighty-first year, are this year to swell the tide of death to boundless eternity, not less than three millions pass on, before their time, their own suicides?

I propose to show how this waste of human life can be avoided, and how my readers may add many glad years to their existence, except it be their lot to perish by violence. Less than half a dozen words give the requisite instruction; less than half a dozen words contain the almost infallible recipe. *Secure a daily alvine action;* have one motion from the bowels every twenty-four hours. I may say without exception, that nine-tenths of all diseases involve the infraction of this habit. Ask any ten persons coming into a physician's office, if they have one regular daily action of the bowels, and nine of them will answer "*no.*" When a person does not have as many as one action of the bowels during each twenty-four hours, he is said to be "*costive*," to be "*constipated;*" this state of things is "costiveness" or "constipation;" these terms have one and the same meaning. The principle once stated, is self-evident; and yet, perhaps, the majority of men and women who reach the age of twenty-five years, have not felt the necessity of this daily discharge. How many parents who read these lines, can lay them down a moment, and say truly, that they have ever given one lesson to their children as to the importance of attending to it. If you pour into a vessel any amount of water to-day, however small, and repeat the operation daily, that vessel will sooner or later overflow, unless each day as much is let out as was poured in. If you eat a certain amount of food to-day, and nothing passes from the body, it must inevitably become so full in a few days, that you can't swallow any more, that is, nature with her instincts comes to the rescue, and deprives the body of the desire for food; we call it want of appetite; we loathe food, because in reality there is no room for it. This want of appetite is beautifully expressed by the medical term "Anorexia," and in reading any medical work, which describes the symptoms of the various diseases, this word soon becomes an old acquaintance. But let a man who has no appetite, in other words, who has swallowed so much, that he has not room for a morsel more, take an active vomit, take a puke, for I want my most unlearned country friend to understand fully what I mean, and in a few hours he will have the appetite of a horse. Or, if he does not admire the operation of "casting up," he can take a "brisk cathartic," which will relieve his gorged carcass in the opposite direction, and "the premises being evacuated," in law phrase, Richard will be himself again in a day or two. Let the reader understand, that I do not hereby advise him to take a puke or a purge, if he has no appetite, and yet wants one. I am only stating how he may scientifically and promptly recover his appetite, if he has lost it, by allowing constipation. For my own part, I have such an *intestinal abomination* of physic, as Mother Partington would say, that I would rather stay without an appetite for a considerable time, than to take a puke or a purge, especially as I cannot see why any body should want an appetite these times, when beef is eighteen cents a pound, green apples two dollars a bushel, and flour twelve dollars a barrel, such being the prices I have paid in this city within the last ten days. While food is at these prices, money is sky-high; Wall-street says, it is thirty-six per cent. "Under the peculiar circumstances of the case," I would really advise any anorexiated individual to remain in *statu quo*, to repose on his reserved rights. In fact, the man without an appetite now-a-days, is like a traveller without a trunk; he is enviably independent. The conclusion then forces itself upon the understanding, without having had the slightest premeditation in that direction when the heading of this article was penned, before breakfast this morning, that the most direct and prompt cure for the present hard times is to become costive, and then you can snap your finger and thumb triumphantly at butchers, hucksters, green grocers, *et id omne genus*, all that fraternity. But how to become costive, that is a question which comes directly home to the pocket, with cumulative power, because the times, like the ice, are becoming harder every hour.

RECIPES FOR BECOMING COSTIVE.

For yourself, take a little opium, or a few drops of laudanum, which is opium in a liquid form, two or three times a day.

If you want to begin at the beginning, and economize from the baby upward, and make a pint of milk last as long as a quart, give it a little paregoric, (diluted laudanum) every time it cries, or Godfrey's cordial.

If you want next to attack your wife, and anorexiate her, and yet would rather do it on the sly, find out if she has not a little *dryness in the throat*, or a *slight heck or hem*, *or cough*, or a *little clearing of the throat*, you have only to get her one of those nice little boxes filled with any sweetish lozenge, it is perfectly immaterial what name they go by, if it is a lozenge at all, it has the two essential requisites—sugar and opium. No cough lozenge is made which does not contain both these ingredients, and each ingredient acts infallibly in the same direction; the sugar itself, the purest loaf, or the best syrup which can be made, would destroy the tone of the stomach, that is, impair the appetite, if taken "*three times a day before meals*," that being the stereotype recipe for taking all patent medicines. Any thing sweet, thus taken, acts directly on the stomach, and causes want of appetite. Opium causes want of appetite in a more roundabout way, it causes constipation, and that causes loss of appetite, as already explained. Therefore, if sugar alone destroys the appetite, and opium alone does the same thing, both combined, do it in double quick time. I never tasted a lozenge, or a balsam, or balm, or cough mixture, or pectoral, which had not both the sweetish and a bitterish taste, and I presume no one else ever did. An educated druggist would question a man's sanity, who would ask for a cough medicine which had no bitter or sweet taste about it. Therefore, you may set it down as an infallible fact, that no lozenge or cough medicine can be taken even for a short time, without impairing the appetite and causing constipation, that is, preventing a regular daily action of the bowels. There is, however, some caution to be observed in the production of artificial anorexia and constipation; if kept up long in grown persons, a natural and certain result is piles first, and then fistula, which last if cured at all, must be by the surgeon's knife, or my neighbor Bodenhamer will cure your fistula without a knife, but he will expect a fee, ranging from fifty to five hundred dollars. Now, that I have come to count the cost, I think it would be rather a saving after all, to let your wife have her appetite and take no lozenges or cough remedies, so after "*second thoughts*," I would rather advise you never to give or swallow a lozenge or a cough drop as long as you live, unless you wish to be considered a candidate for some lunatic asylum.

As for the baby, it likes anything sweet, at least my Bob, and our new little Alice glory in sweets, and as they are but a type of their kind, I conclude that all children like anything sweetish, and they will take the lozenge or the "syrup" from the father's or the mother's hand, with such loving, smiling confidence, that one must smile and love in return to witness it. It is true, these things do, in a few weeks, give by degrees an unusual brightness of the eye, succeeded by water on the brain on the first attack of sickness, and all its growth is in the head, and its little body dwindles, and its eyes stare out with a maniacal phrensy or an idiotic blankness, closing soon in death, but then, you have saved a pint of milk a day for a good while.

What I have written refers to scientific constipation. I began the article with the intention of explaining simply how persons generally became costive, and no more important explanation in reference to bodily health has ever appeared in the pages of this journal nor ever will. The answer to the question,

HOW DO PERSONS GENERALLY BECOME COSTIVE?

I do not recollect to have seen in any publication, popular or professional that I have ever read, and yet it will come home to every thinking reader. It is of authenticated and historical record that in the last war between China and Great Britain, the Chinese confidently anticipated ultimate victory by negative means alone; it is almost incredible, and yet it is a fact that they believed that if they cut off the supplies of rhubarb, the British would all die, because that article is known to be used to prevent constipation, and if it could not be had, the British soldiers would bloat up and explode, or at least die in consequence; it cannot be denied that constipation would conquer Sebastopol sooner far than the allied army. It is very certain that the lozenges and cough medicines in New York, if they were taken by the Russians, would end the siege of the consecrated city sooner than will be done by the combined forces of the Gallic Eagle and the British Lion.

HOME ILLUSTRATION.

To explain the effects of constipation upon human health and life by objects nearer to us than the Crimea, take a steam engine; if the steam is not worked off as fast as it accumulates in the boiler, total destruction is absolutely inevitable. The smallest particles of dust will one by one find their way from the vest pocket into your watch, and in a year or two, the accumulation will have been such, that the whole machinery is clogged and it stands still, and so with the clock on your mantel, however closely it may be shut and covered every time your tidy house-keeper "dusts the room." It is because there is a constant inlet, yet no outlet, and just as certainly, just as inevitably will the machinery of life stand still, sooner or later if we eat daily and do not pass from us as daily, the refuse of what we eat, after it has subserved the purposes of life. If what we eat to-day, and its refuse, does not pass from us to-morrow, it remains but to clog, and irritate, and inflame, and fester and destroy, and rot every part with which it comes in contact.

How then do persons generally become costive? How does the young woman pine away before maturity? How does the strong young man, who almost thinks that nothing can hurt, wither and waste and die long before his prime? How is it that the mass of men do not live out half their days? These questions are all answered by stating the manner in which the regular functions of the bowels are deranged.

Order is Heaven's first law. Regularity is nature's universal rule. Morning, noon, and night, the healthy man becomes hungry at the usual eating hour for half a century, no human machine can work the twentieth part so long without adjustment or repair; at the accustomed hour the infant becomes sleepy; within ten minutes of the time does the regular man wake of a morning for weeks and months in succession. So is it with the desire to stool: with almost all it comes on soon after breakfast, this appears to be the most proper time, and if not interfered with, this inclination will come on for a life time with but a few minutes variation, and a healthful old age is the result, but if interfered with, the foundation begins to be laid of nine-tenths of all our maladies, and a premature and painful death. And here we come to the most important item in this article:

HOW IS THE DAILY ACTION OF THE BOWELS INTERFERED WITH?

Reader, I will appeal to your own experience, confident that millions of others would respond to it if questioned. I will suppose you to have good health, that usually after breakfast awhile you experience an inclination to go to the privy, generally you do go promptly, but sometimes you do not, you are reading an interesting newspaper article, and you went to finish it, or a chapter of a novel, or a political speech, or scientific lecture, or are attending to an early visitor, hoping every moment his departure, or you are hemming a handkerchief, or engaged on a piece of embroidery, or you are hurried down town by inexorable business, and when the desire comes, there is no convenient locality. I might mention dozens more of instances which are presented as inducements to defer nature's demand for the moment, and before you are aware of it, the desire has departed, and hours may elapse before it is felt again, and so faintly that absorption in business, may prevent its notice; the next day it comes later and fainter, and before you are aware of it, you have fallen into the habit of passing a day or two or more without attending to a call of nature; and the next thing you observe the symptoms of some troublesome disease, an illustration of which I now give in order to impress upon the reader's attention the evils which may result from constipation.

A British soldier was wounded in the Spanish war at Barossa, in 1811, and having served twenty-one years in the army, he was placed on the pension list, which he enjoyed for forty-one years in sound health; but lately, on leaving work, he became liable to constipation. At first, his bowels moved every other day, then seldom oftener than once a week, and finally only once in four weeks; at last, his belly became so large that his trowsers would not meet, and he applied to Professor Christison to enable him to button his breeches; he measured at the waistband near forty inches. The proper means were used to procure a discharge, and an immense amount was the result; on other medicine being administered, another immense discharge was the result; still his belly was as large as ever, and next day a third dose of medicine was exhibited, which gave an ordinary discharge, and on the third day, there being no diminution in size, two tea-spoons of turpentine and twelve table-spoons of castor oil gave only two small passages, and the abdomen was as large as ever; extreme and painful means were then used with more success, but he declared with an oath he never would submit to them, and had rather be shot; but being allowed a day's rest, he did submit next day, and at the end of a fortnight's treatment he was dismissed with daily-acting bowels, in his seventy-fourth year.

The great practical lesson which I wish to inculcate, to be engraven, as on a plate of steel, on the memory of children and youth, young men and women, the mature and the gray-headed :—☞ *Allow nothing short of fire or endangered life to induce you to resist for one single moment nature's alvine*

*call.* So far from repressing a call for any reason short of life and death, you should go at the usual time and solicit, and doing so, you will have your reward in a degree of healthfulness and in a length of life which very few are ever permitted to enjoy.

If the love of health and life, nor the fear of inducing painful disease cannot induce you to adopt the plan I have recommended, there is another argument which to young gentlemen and young ladies may appear more convincing, personal cleanliness.

If you resist a call of nature, a degree of uneasiness and irritation and heat is the immediate result; this heat causes the more airy and watery particles of the fecal matter, which is waiting to be discharged, to evaporate and to be re-absorbed into the system, to be taken into the blood again, which bears the horrible burden to the lip of beauty, which we kiss with so much devotion; and the very tear-drop of affection has mingled with it what ought to have been deposited in the privy a few hours before, making the very breath unbearably disgusting: the breath of a costive child even is scarcely to be endured.

Cold feet, sick head-ache, piles, fistulas, these with scores of other diseases have their first foundations laid in constipation, which itself is infallibly induced by resisting nature's first calls. Reader, let it be your wisdom, never to do it again.

